# Amputation by the Ecraseur

**Published:** 1869-11

**Authors:** 


### *Amputation by the Ecraseur.*

M. Bardinet, of Limoges, in the *Bulletin Général Thérapeutique.* for June 15th, recommends the *Ecraseur* as a means of performing amputation of the limbs, and states that he has in this way removed the leg. In operating, he first cuts through the skin, and then divides the tissues, in separate portions, by the *Ecraseur*, from without inward. Lastly, the bone is sawn through. M. Chassaignac in a note to the editor of the *Bulletin*, says that, though he has used the *Ecraseur* successfully in two cases of amputation of the thigh, he does not consider this instrument suitable for amputations in general, inasmuch as it is not capable of ready application in all cases.—*N. Y. Med. Jour.*

